# Gastrointestinal Parasites With Their Risk Factors in Tharu Indigenous People in Southern Nepal: A Cross‐Sectional Study

**DOI:** 10.1002/hsr2.70385

**Published:** 2025-01-27

**Authors:** Pinki Kumari Chaudhary, Tirth Raj Ghimire

**Affiliations:** ^1^ Department of Zoology, Tri‐Chandra Multiple Campus Tribhuvan University Kathmandu Nepal

**Keywords:** cryptosporidium, gastrointestinal parasites, Tharu people

## Abstract

**Background:**

Globally, gastrointestinal (GI) infections are common, particularly in populations with low socioeconomic levels, including high illiteracy rates, ignorance, poor housing and lifestyles, and unfavorable environmental conditions. These risk factors are the underlying cause of GI parasitic infections in many developing nations, including Nepal.

**Aim:**

This study aimed to assess the prevalence and diversity of GI parasites and their association with a few risk factors within the marginalized indigenous Tharu people of the southern region of Nepal.

**Methods:**

A purposive sampling method was used to collect stool samples from Tharu indigenous people (*N* = 179) in Thaskaul, Kolhabi, Bara, Nepal. The samples were preserved in a 2.5% potassium dichromate solution. The samples were analyzed by direct wet mount and acid‐fast staining techniques and examined at 40×, 100×, 400×, and 1000× magnifications.

**Results:**

Out of 179 stool samples, the prevalence rate of the GI parasites was 42.46% (*n* = 76), out of which females (43.00%, 43/100) had a slightly higher prevalence rate than males (41.77%, 33/79) (*p* > 0.05, chi‐square tests at 95% confidence level). Out of nine species of GI parasites, the prevalence of *Cryptosporidium* spp. was highest among protozoa (30.17%, *n* = 54), whereas that of *Ascaris lumbricoides* was highest among helminths (5.59%, *n* = 10). Occupation, animal husbandry, parasitologic knowledge, drinking water sources, pork‐feeding habit, diarrheal or stomachache symptoms, and drug‐consuming history were associated with one or more GI parasitosis (*p* < 0.05).

**Conclusion:**

The moderate prevalence and diversity of GI parasitism in the Tharu population suggests the need for effective, efficient, and timely preventative and control measures in the study area. Further One Health Approach, by conducting studies that involve the examination of samples from the local populations, livestock, food, water, and soil, would be important.

## 1 Introduction

Nepal is a multiethnic country in the lap of the Himalayas, where 142 castes/ethnicities with 124 mother tongues are situated in a perfectly coherent society [1]. Among these ethnic/caste groups, Tharu is historically, culturally, and socially significant in Nepal and several parts of India. Tharu has a total of 18,07,124 population, comprising 6.2% of the total population in Nepal [1]. They are interesting to parasitologists because of gastrointestinal (GI) parasite dominance in different studies [2, 3, 4], indicating the significance of further studies.

GI parasitic infections are common everywhere, but mainly in poor and undeveloped nations, where their prevalence rate ranges from 90% to 95% [5]. GI parasitic infections are mainly caused by helminths like *Ascaris lumbricoides, Ancylostoma, Strongyloides, Trichuris, Enterobius*, and protozoa like *Balantidium, Cryptosporidium, Cyclospora, Entamoeba*, and *Giardia*. These parasites are predominant around the world [5]. They can cause anemia, diarrhea, nutritional deficiency, conjunctivitis, bloody sputum, intestinal obstruction, respiratory complications, neurological complications, and retarded mental and physical growth leading to significant morbidity and mortality [6, 7, 8, 9, 10, 11, 12, 13, 14, 15, 16]. Notably, the GI parasitic infections have been evidenced to be associated with consumption of contaminated drinking water, lack of toilets, ignorance, unhygienic eating habits, poor sanitation, low economic status, and unfavorable personal and environmental conditions [2, 17, 18, 19]. Even though these risk factors dominate in Nepal, its government has tried eradicating parasitosis through many deworming programs by collaborating with the World Health Organization (WHO) and several other organizations for many years. For example, the government distributed vitamin A capsules and antiparasitic drugs to children of 12 months to 59 months twice a year in a few districts in 2000 and then scaled up all over the country by 2010 [20]. The supplementation of vitamin A is due to its beneficial roles in obstacle to parasites, anti‐inflammatory, and T cell, B cell, and cytokine responses [21, 22, 23].

Previous studies have shown GI parasitic problems in the Tharu population in southern and central Nepal [2, 3, 4], and several risk factors might exist for GI parasitosis in this population. A pilot survey before this study recorded the need for proper awareness regarding parasitic infections, lack of education, and drinking water and toilet facilities in the Thaskaul, Kolhabi Municipality in southern Nepal. In addition, feeding on improperly cooked pork might increase tapeworm (*Taenia* spp.) infections, as pigs are critical intermediate hosts. These risk factors play roles in GI parasite transmission. However, none of the parasitological studies have been conducted in the Tharu population in this Municipality. Therefore, this study aimed to investigate the prevalence and diversity of GI parasites and their probable risk factors in the Tharu community in southern Nepal.

## 2 Materials and Methods

### 2.1 Study Design

A descriptive cross‐sectional study was conducted on the local Tharu people in the Thaskaul area, Kolhabi Municipality‐11, Bara, Nepal, from 9 November 2022 to 30 March 2023.

### 2.2 Study Area and Participants

**Figure 1:**
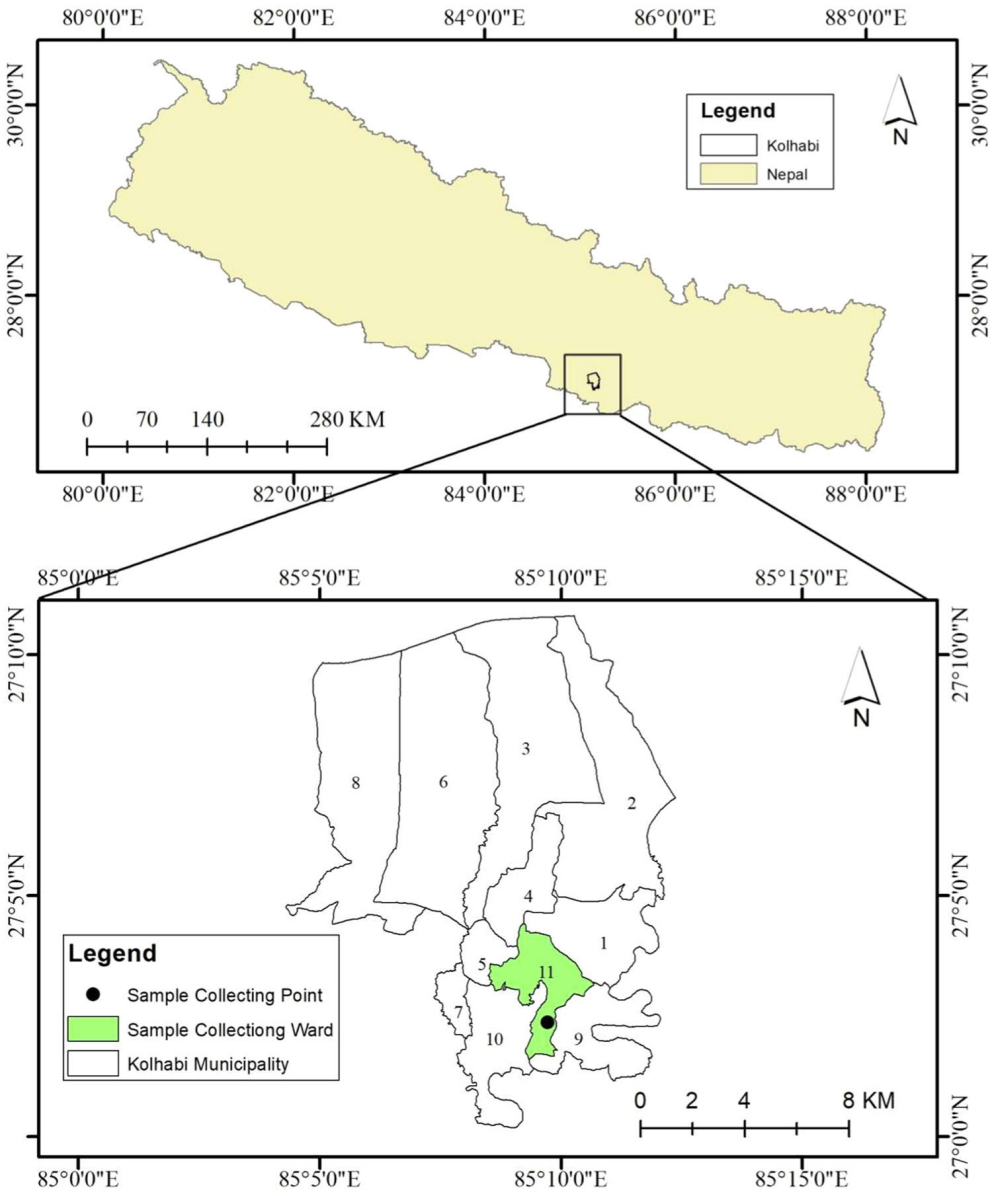
Study area map (Arc Map 10.8, ArcGIS Desktop software package 10.8, 2019).

The Kolhabi Municipality Ward Number 11 in the Thaskaul area was selected for the study as none of the studies were previously conducted (Figure 1). Municipality is surrounded by Rautahat district in the east, Jitpur Simara Sub Metropolitan City, Kalaiya Sub Metropolitan City, Karaiyamai Rural Municipality in the west, Nijgadh Municipality in the North, and Gadhi Rural Municipality and Karaiyamai Rural Municipality in the south. It is approximately 126 kilometers to the southern part of Nepal, away from Kathmandu, the capital city, by the shortest highway route.

Ward number 11 possesses 3332, and the Thaskaul area possesses 1060 total individuals of 18 years and older than that age (https://kolhabimun.gov.np/en, accessed on 27 April 2024). Most of them are Tharu people, although other groups like Brahmin, Majhi, Musahar, and Thakur live there. Their primary occupation is farming, agriculture, and rearing livestock; however, a few people have driving, housework, health work, study, and business. Their diet includes meat, mostly snails, crabs, pigeons, fish, prawns, freshwater clams, pork, chicken, and mutton. However, few of these people do not consume pork and other types of meat.

### 2.3 Inclusion Criteria

The study included Tharu individuals aged 18 years and older who were mentally and physically strong.

### 2.4 Sample Size Calculation

From 9 November 2022 to 30 March 2023, a purposive sampling method was used to study endoparasitic infections among 179 people (18 years or more), representing about 17% of the Thaskaul area and > 5% of the total ward population.

### 2.5 Exclusion Criteria

Individuals who did not want voluntary participation, were mentally and physically weak, and were less than 18 years old were not involved in the study. Also, individuals other than Tharu people were excluded from the study.

### 2.6 Socio‐Demographic and Behavioral Data Collection

An informed consent was obtained from each participant. A questionnaire with information on age, sex (biologically male or female), occupation, education levels, sources of drinking water, drinking habits, levels of environmental status, defecation habits, animal husbandry, pork feeding habit, knowledge on GI parasites, experienced symptoms (stomachache or diarrhea), GI illness procedures, and consumed drug duration with informed consent was designed and distributed to the participants based on previous researches [2, 4, 17]. An interview was taken if the participant could not write it down.

### 2.7 Sample Collection and Transportation

After the questionnaire and interview survey, a 30 milliliter (mL) sterile vial was distributed so each participant could collect the stool sample early the following day. Each participant's stool samples in the morning were collected in the 30 mL vial. The stool samples were initially macroscopically examined for blood, mucus, adult worms or their segments. Then, the samples were preserved in a 2.5% potassium dichromate solution. The samples were transported to the Zoology Laboratory (Tri‐Chandra Multiple Campus, Tribhuvan University, Kathmandu, Nepal).

### 2.8 Sample Processing and Examination

The samples were processed using the wet mount and acid‐fast techniques as standardized previously [9, 11, 24, 25].

#### 2.8.1 Wet Mount Techniques

About 2 grams (gms) of the fecal sample were stirred/mixed carefully for direct wet mount. A single drop of each sample was put on the glass slide with or without Gram's iodine stain. Then, by covering the sample with a coverslip, it was observed under a microscope at a total magnification of 40×, 100×, and 400×.

#### 2.8.2 Modified Acid‐Fast Staining Technique

For the modified acid‐fast staining technique, the sediments obtained following the formal‐ether method (1200 rpm × 5 min at 10 mL of 10% formalin and 3 mL of ethyl acetate) were used to prepare thin smears. The dried smears were fixed in absolute methanol for 2 min, stained with carbol fuchsin for 15 min, washed with acid alcohol and distilled water for 2 min. The dried smear was examined under a compound microscope at 1000× over immersion oil, especially for observing coccidian oocysts.

### 2.9 Identification of Intestinal Parasites

Photographs of all the stages of the parasites (cysts, oocysts, trophozoites, eggs, and larvae) observed were captured. The parasites were identified using the methodology given in the literature [6, 10, 17].

### 2.10 Data Analysis

Data were present on tables in Microsoft Word 2007 and bar charts on GraphPad Prism (Version 5.00). Mean, standard deviation, percentage, and descriptive graphs were determined for a particular parasite or its groups. The association of bivariate characteristics of a risk factor with total GI parasites or particular GI parasite was analyzed by Fisher's exact tests (Two‐sided) with relative risk (RR) (95% confidence interval), odds ratio (OR) (95% confidence interval), and likelihood ratio (LR). Multiple variables within the same risk factors were analyzed by the Chi‐square test for trend unless otherwise stated. Data were considered significant in both cases if probability (*p*)‐values were less than 0.05. All statistical analyses were performed using GraphPad Prism (Prism 5 for Windows Version 5.00, 2007).

## 3 Results

### 3.1 Prevalence and Diversity of GI Parasites

**Figure 2:**
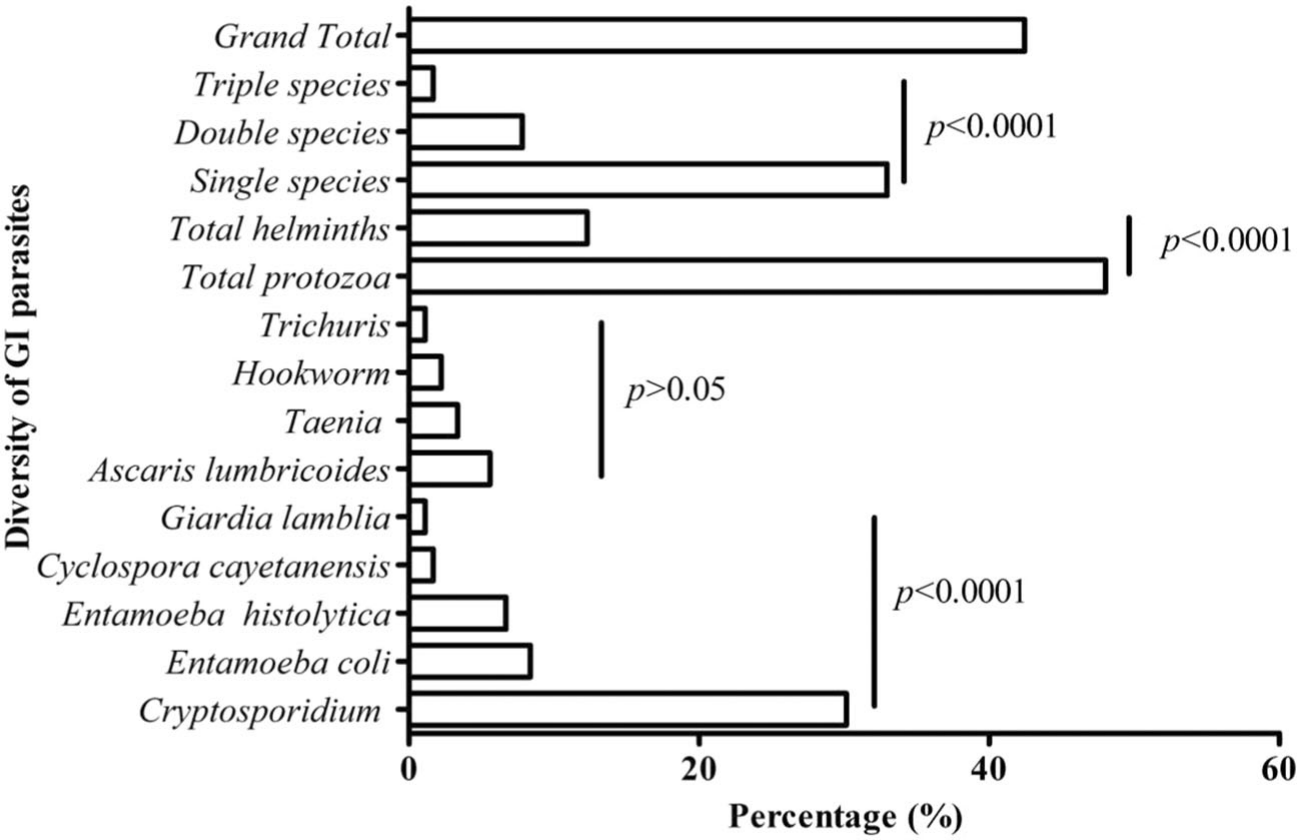
Percentage (%) of different GI parasites. The *p* values were obtained by the Chi‐square tests (two‐tailed) compared among the parasites or groups represented by lines.

**Figure 3:**
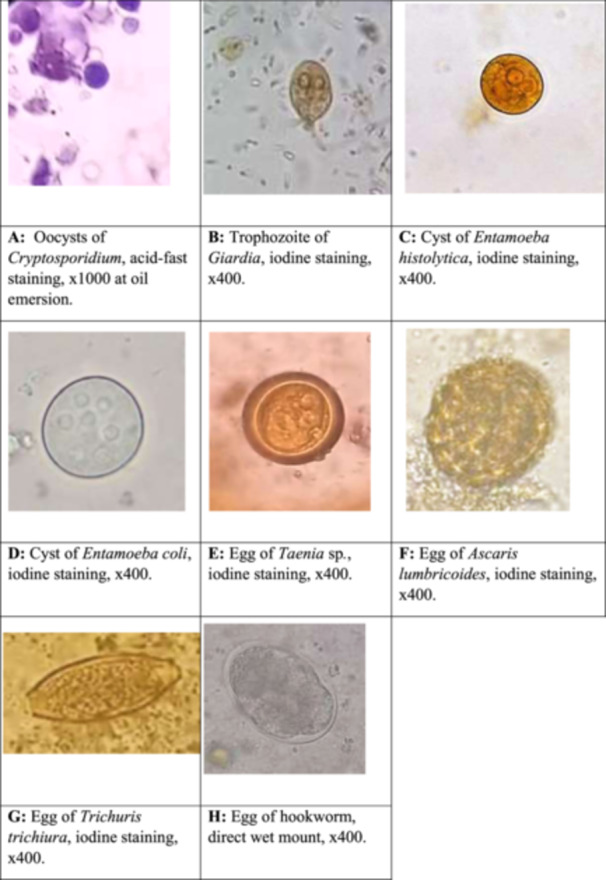
Oocyst, trophozoite, cyst, and egg of GI parasites. (A) Oocysts of *Cryptosporidium*, acid‐fast staining, ×1000 at oil emersion. (B) Trophozoite of *Giardia*, iodine staining, ×400. (C) Cyst of *Entamoeba histolytica*, iodine staining, ×400. (D) Cyst of *Entamoeba coli*, iodine staining, ×400. (E) Egg of *Taenia* sp., iodine staining, ×400. (F) Egg of *Ascaris lumbricoides*, iodine staining, ×400. (G) Egg of *Trichuris trichiura*, iodine staining, ×400. (H) Egg of hookworm, direct wet mount, ×400.

Out of 179 stool samples in this study, 76 (42.45%) were positive for GI parasites. Similarly, different prevalence rates of nine diverse parasites with five protozoa (*Cryptosporidium* spp., *Entamoeba coli*, *E. histolytica, Cyclospora cayetanensis*, and *Giardia lamblia*) (*p* < 0.0001) and four helminths (*Ascaris lumbricoides*, *Taenia* spp., hookworms, and *Trichuris trichiura*) (*p* > 0.05) were detected. Out of nine species, the prevalence of *Cryptosporidium* spp. was highest among protozoa (30.17%, *n* = 54), whereas that of *Ascaris lumbricoides* was highest among helminths (5.59%, *n* = 10). Interestingly, the prevalence of protozoan species was about four times higher than helminths (*p* < 0.0001). Similarly, the prevalence of stool samples with single infection (32.96%) was higher than the duplet (7.82%) or triplet (1.68%) infections (*p* < 0.0001) (Figures 2 and 3).

### 3.2 Sex‐Wise Prevalence and Diversity of GI Parasites

Then, the sex‐wise prevalence of GI parasites was evaluated to analyze whether male and female have different parasitic diversity and prevalence. Females had a slightly higher prevalence rate than males had (43.00%, 43/100 vs. 41.77%, 33/79) (*p* > 0.05). The prevalence rates of *Cryptosporidium* spp., *Cyclospora cayetanensis*, hookworm, and *Trichuris trichiura* were higher in females compared to males without any statistical significance (*p* > 0.05). Similarly, *Entamoeba coli, E. histolytica, Giardia lamblia*, *Ascaris lumbricoides*, and *Taenia* spp., were higher in males than females; however, the data were statistically nonsignificant (*p* > 0.05). However, analysis of OR, RR, and LR indicated that the males were more likely to be infected with *Taenia* spp. than females. A total protozoan, helminth, and both groups were more prevalent in males than females (*p* > 0.05). As assessed by the number of species, concomitance of parasites showed that females had a high prevalence of single and double infection (*p* > 0.05), but triple infection was present only in males (Table 1).

**Table 1:** Prevalence (%) of different gastrointestinal (GI) parasites. The *p* values (Fisher's exact tests), relative risk (RR), odds ratio (OR), and likelihood ratio (LR) were assessed comparing particular parasitic species, groups, or concomitance between males and females.

Parasitic diversities	No. (%) of males (n1 = 79)	No. (%) of females (n2 = 100)	*p* values	RR (95% CI); OR (95% CI); LR
**Protozoan diversities**				
*Cryptosporidium* spp.	23 (29.11)	31 (31)	> 0.05	0.939 (0.598–1.475); 0.914 (0.480–1.742); 0.952
*Entamoeba coli*	9 (11.39)	6 (6)	> 0.05	1.900 (0.705–5.11); 2.014 (0.685–5.92); 1.406
*E. histolytica*	7 (8.86)	5 (5)	> 0.05	1.772 (0.584–5.370); 1.847(0.563–6.060); 1.353
*Cyclospora cayetanensis*	1 (1.26)	2 (2)	> 0.05	0.633 (0.058–6.858); 0.628 (0.056–7.061); 0.752
*Giardia lamblia*	1 (1.26)	1 (1)	> 0.05	1.267 (0.080–19.930); 1.269 (0.078–20.630); 1.135
**Helminthic diversities**				
*Ascaris lumbricoides*	5 (6.33)	5 (5)	> 0.05	1.266 (0.380–4.22); 1.284 (0.358–4.602); 1.142
*Taenia* spp.	5 (6.33)	1 (1)	> 0.05	6.329 (0.754–53.11); 6.689 (0.765–58.50); 1.948
Hookworm	1 (1.26)	3 (3)	> 0.05	0.422 (0.045–3.981); 0.415 (0.042–4.066); 0.560
*Trichuris trichiura*	0 (0.0)	2 (2)	> 0.05	0.000 (Infinity); 0.248 (0.012–5.24); 0.000
**Total protozoa**	34 (43.04)	40 (40)	> 0.05	1.076 (0.759–1.526); 1.133 (0.623–2.063); 1.072
**Total helminths**	11 (13.92)	11 (11)	> 0.05	1.266 (0.579–2.767); 1.309 (0.536–3.199); 1.154
**Total positive**	45 (56.96)	51 (51)	> 0.05	1.117 (0.851–1.465); 1.272 (0.702–2.302); 1.144
**Concomitance**				
One species	25 (31.65)	34 (34)	> 0.05	0.931 (0.609–1.422); 0.899 (0.479–1.686); 0.942
Two species	5 (6.33)	9 (9)	> 0.05	0.703 (0.245–2.015); 0.683 (0.219–2.127); 0.796
Three species	3 (3.79)	0 (0.0)	> 0.05	9.200 (0.468–180.8); 2.316

### 3.3 Age‐Wise Prevalence and Diversity of GI Parasites

In this study, the mean and SD of the studied population (*N* = 179) ranging from 18 to 80 years was 38.58 and 14.09, respectively. Age‐wise prevalence of *Cryptosporidium* spp. (*p* > 0.05), *E. coli* (*p* < 0.05), total protozoa (*p* > 0.05), total positive (*p* > 0.05), and double parasitic species (*p* > 0.05) showed the highest in the 18–40 year age group compared to the others. Similarly, *E. histolytica* (*p* > 0.05), *Cyclospora cayetanensis* (*p* > 0.05), hookworm (*p* > 0.05), and triple parasitic species (*p* > 0.05) were higher in the age groups 41–60 years. *Giardia lamblia* (*p* > 0.05), *Taenia* sp. (*p* > 0.05), total helminths (*p* > 0.05), and single infections (*p* > 0.05) were highest in people of the 61–80 years of age group (Table 2).

**Table 2:** Age‐wise prevalence (%) of GI (gastrointestinal) parasites. The *p* values (Chi‐square by trends) were assessed comparing particular parasitic species, group, or concomitance among three different age‐groups. SD: Standard Deviation.

Parasitic diversities	No. (%) (18–40 yrs) (n1 = 116)	No. (%) (41–60 yrs) (n2 = 45)	No. (%) (61–80 yrs) (n3 = 18)	*p* values	Mean; SD
**Protozoan diversities**					
*Cryptosporidium*	40 (34.48)	9 (7.76)	5 (27.77)	> 0.05	36.43; 13.59
*Entamoeba coli*	13 (11.20)	2 (4.44)	0 (0.0)	< 0.05	31.87; 7.57
*Entamoeba histolytica*	7 (6.03)	4 (8.89)	1 (5.56)	> 0.05	39.63; 14.20
*Cyclospora cayetanensis*	0 (0.0)	3 (6.66)	0 (0.0)	> 0.05	35.45; 10.39
*Giardia lamblia*	1 (0.86)	0 (0.0)	1 (5.55)	> 0.05	49.75; 29.34
**Helminthic diversities**					
*Ascaris lumbricoides*	4 (3.448)	4 (8.88)	2 (11.11)	> 0.05	45.9; 16.46
*Taenia* sp.	5 (4.31)	0 (0.0)	1 (5.55)	> 0.05	35.92; 16.94
Hookworm	2 (1.724)	2 (4.44)	0 (0.0)	> 0.05	39.75; 12.41
*Trichuris trichiura*	2 (1.72)	0 (0.0)	0 (0.0)	> 0.05	29; 0
**Total protozoa**	54 (46.55)	14 (31.11)	6 (33.33)	> 0.05	36.43; 13.20
**Total helminths**	13 (11.20)	6 (13.33)	3 (16.67)	> 0.05	40.52; 15.46
**Total positive**	67 (57.75)	20 (44.44)	9 (50)	> 0.05	37.37; 13.77
**Concomitance**					
One species	37 (31.89)	13 (28.88)	9 (50)	> 0.05	40.07; 15.69
Two species	12 (10.34)	2 (4.44)	0 (0.0)	> 0.05	32.07; 7.8
Three species	2 (1.72)	1 (2.22)	0 (0.0)	> 0.05	36.17; 12.411

### 3.4 Risk Factors and Diversity of GI Parasites

The association of all or particular GI parasite species in the fecal samples was analyzed based on different respondents' characteristics. The drug consumption rate was lower than nonconsumption (106, 59.22%), and GI parasites were higher in the nonconsumers; however, there was no statistical significance (*p* > 0.05). The risk was again calculated for those individuals (73, 40.78%) who had historically taken antiparasitic medicines before 1 month to more than 12 months. Interestingly, parasitologic knowledge (*p* < 0.05), diarrhea or stomachache (*p* < 0.005), and drug‐consuming history (*p* < 0.05) were associated with total GI parasites. Occupation, animal husbandry, pork‐feeding habit, and clinical symptoms like diarrhea or stomachache were associated with *Cryptosporidium* spp. (*p* < 0.05). Occupation, drinking water sources, and drug‐consuming history were associated with *E. coli* (*p* < 0.05). Occupation and drinking water sources were associated with *Taenia* sp. (*p* < 0.05). Animal husbandry (*p* < 0.05) and drug‐consuming history were strongly associated with *Ascaris lumbricoides* (*p* < 0.05). Drug‐consuming history was also associated with *E. histolytica* (*p* < 0.05) and hookworm (*p* < 0.0001). A strong association based on RR, OR, and LR was also observed in *Cryptosporidium*‐ or *Ascaris lumbricoides*‐positive individuals who had animal husbandry in their houses or who used to consume pork (Table 3).

**Table 3:** Prevalence (%) and risk factors of various GI parasites in the Tharu community in southern Nepal. Bivariate analysis was done using Fisher's exact tests to calculate *p* values, relative risk (RR), odds ratio (OR), and likelihood ratio (LR). Multivariate analysis was done using Chi‐square by trends to calculate *p* values.

Respondent's characteristics			Number	Positive	*Cryptosporidium*	*E. coli*	*E. histolytica*	*Cyclospora*	*Giardia*	*Taenia*	*Ascaris*	Hookworm	*Trichuris*
**Occupation**	Farmer		57	22 (38.6)	11 (19.3)	2 (3.5)	4 (7.0)	2 (3.5)	1 (1.8)	0 (0)	5 (8.8)	3 (5.3)	0 (0)
	Student		62	23 (37.1)	14 (22.6)	3 (4.8)	1 (1.6)	0 (0)	1 (1.6)	1 (1.6)	3 (4.8)	1 (1.6)	1 (1.6)
	Housewife		31	18 (58.1)	18 (58.1)	5 (16.1)	3 (9.7)	0 (0)	0 (0)	2 (6.5)	0 (0)	0 (0)	1 (3.2)
	Businessman		22	11 (50)	10 (45.5)	5 (22.7)	4 (18.2)	0 (0)	0 (0)	3 (13.6)	1 (4.6)	0 (0)	0 (0)
	Teacher		6	1 (16.7)	1 (16.7)	0 (0)	0 (0)	1 (16.7)	0 (0)	0 (0)	0 (0)	0 (0)	0 (0)
	Driver		1	1 (100)	0 (0)	0 (0)	0 (0)	0 (0)	0 (0)	0 (0)	1 (100)	0 (0)	0 (0)
***p* values**				> 0.05	< 0.05	< 0.05	> 0.05	> 0.05	> 0.05	< 0.05	> 0.05	> 0.05	> 0.05
**Education status**		Illiterate	37	20 (54.1)	12 (32.4)	1 (2.7)	4 (10.8)	2 (5.4)	1 (2.7)	1 (2.7)	4 (10.8)	2 (5.4)	0 (0)
		Literate	142	56 (39.4)	42 (29.6)	14 (9.9)	8 (5.6)	1 (0.7)	1 (0.7)	5 (3.5)	6 (4.2)	2 (1.4)	2 (1.4)
***p* values**				> 0.05	> 0.05	> 0.05	> 0.05	> 0.05	> 0.05	> 0.05	> 0.05	> 0.05	> 0.05
**Drinking water sources**		Hand pipe	164	71 (43.3)	49 (29.6)	11 (6.7)	9 (5.5)	3 (1.8)	2 (1.2)	3 (1.8)	10 (6.1)	4 (2.4)	2 (1.2)
		Jar (Bottled)	15	5 (33.3)	5 (33.3)	4 (26.7)	3 (20)	0 (0)	0 (0)	3 (20)	0 (0)	0 (0)	0 (0)
***p* values**				> 0.05	> 0.05	< 0.05	> 0.05	> 0.05	> 0.05	< 0.05	> 0.05	> 0.05	> 0.05
**RR (95% CI), OR (95% CI), and LR**						0.252 (0.091–0.694); 0.198 (0.054–0.724); 0.786				0915 (0.020–0.414); 0.074 (0.014–0.410); 0.537			
**Water treatment**		Yes	5	0 (0)	0 (0)	0 (0.0)	0 (0)	0 (0)	0 (0)	0 (0)	0 (0)	0 (0)	0 (0)
		No	174	76 (43.7)	54 (31.0)	15 (8.6)	12 (6.9)	3 (1.7)	2 (1.1)	6 (3.4)	10 (5.7)	4 (2.3)	2 (1.2)
***p* values**				> 0.05	> 0.05	> 0.05	> 0.05	> 0.05	> 0.05	> 0.05	> 0.05	> 0.05	> 0.05
**Levels of environmental status**		Best	22	7 (31.2)	7 (31.8)	5 (22.7)	3 (13.6)	1 (4.5)	0 (0)	3 (13.6)	0 (0)	0 (0)	0 (0)
		Good	146	63 (43.2)	45 (30.8)	10 (6.8)	7 (4.7)	2 (1.4)	2 (1.4)	2 (1.4)	10 (6.8)	4 (2.7)	2 (1.4)
		Worse	11	6 (54.5)	2 (18.2)	1 (9.1)	2 (18.2)	0 (0)	0 (0)	1 (9.1)	0 (0)	0 (0)	0 (0)
***p* values**				> 0.05	> 0.05	> 0.05	> 0.05	> 0.05	> 0.05	> 0.05	> 0.05	> 0.05	> 0.05
**Defection habits**		Open	53	23 (43.4)	18 (34.0)	2 (3.8)	2 (3.8)	1 (1.9)	1 (1.9)	0 (0)	1 (1.9)	1 (1.9)	0 (0)
		Toilet	126	53 (42.1)	36 (28.6)	13 (10.3)	10 (7.9)	2 (1.6)	1 (0.8)	6 (4.8)	9 (7.1)	3 (2.4)	2 (1.6)
***p* values**				> 0.05	> 0.05	> 0.05	> 0.05	> 0.05	> 0.05	> 0.05	> 0.05	> 0.05	> 0.05
**Animals husbandry**		Yes	117	54 (46.2)	42 (35.9)	10 (8.5)	8 (6.8)	3 (2.6)	2 (1.7)	5 (4.3)	10 (8.5)	4 (3.4)	2 (1.7)
		No	62	22 (35.5)	12 (19.4)	5 (8.1)	4 (6.5)	0 (0)	0 (0)	1 (1.6)	0 (0)	0 (0)	0 (0)
***p* values**				0.2042	< 0.05	> 0.05	> 0.05	> 0.05	> 0.05	> 0.05	< 0.05	> 0.05	> 0.05
**RR (95% CI), OR (95% CI), and LR**					1.856 (1.056–3.257); 2.333 (1.119–4.865); 1.296						‐12.21(0.7028–212.1); 1.579		
**Pork‐feeding habit**		Yes	88	39 (44.3)	34 (38.6)	10 (11.4)	8 (9.1)	1 (1.1)	1 (1.1)	4 (4.5)	6 (6.8)	2 (2.3)	0 (0)
		No	91	37 (40.7)	20 (22.0)	5 (5.5)	4 (4.4)	2 (2.2)	1 (1.1)	2 (2.2)	4 (4.4)	2 (2.2)	2 (2.2)
***p* values**				> 0.05	< 0.05	> 0.05	> 0.05	> 0.05	> 0.05	> 0.05	> 0.05	> 0.05	> 0.05
**RR (95% CI), OR (95% CI), and LR**					1.758 (1.101–2.808); 2.235 (1.160–4.308); 1.457								
**Knowledge on GIP**		Very little	88	28 (31.8)	21 (23.9)	5 (5.7)	5 (5.7)	1 (1.1)	1 (1.1)	2 (2.3)	2 (2.3)	1 (1.1)	1 (1.1)
		Nothing	91	48 (52.7)	33 (36.3)	10 (11.0)	7 (7.7)	2 (2.2)	1 (1.1)	4 (4.4)	8 (8.8)	3 (3.3)	1 (1.1)
***p* values**				< 0.05	> 0.05	> 0.05	> 0.05	> 0.05	> 0.05	> 0.05	> 0.05	> 0.05	> 0.05
**RR (95% CI), OR (95% CI), and LR**				0.603 (0.420–0.867); 0.418 (0.227–0.769); 0.633									
**Diarrhea/Stomachache**		Yes	84	41 (48.8)	36 (42.9)	11 (13.1)	9 (10.7)	2 (2.4)	1 (1.2)	3 (3.6)	4 (4.8)	3 (3.6)	2 (2.4)
		No	95	35 (36.8)	18 (18.9)	4 (4.2)	3 (3.2)	1 (1.1)	1 (1.1)	3 (3.2)	6 (6.3)	1 (1.1)	0 (0)
***p* values**				0.1300	< 0.005	0.05	> 0.05	> 0.05	> 0.05	> 0.05	> 0.05	> 0.05	> 0.05
**RR (95% CI), OR (95% CI), and LR**					2.262 (1.394–3.669); 3.208 (1.640–6.276); 1.736	3.110 (1.029–9.403); 3.428 (1.048–11.22); 1.647							
**GI illness treatment procedures**		Hospitals	151	61 (40.4)	47 (31.1)	11 (7.3)	9 (6.0)	2 (1.32)	2 (1.3)	5 (3.3)	10 (6.6)	4 (2.6)	2 (1.3)
		Medicals	15	6 (40)	4 (26.7)	1 (6.7)	1 (6.7)	1 (6.7)	0 (0)	0 (0)	0 (0)	0 (0)	0 (0)
		Hospitals + witch doctors	13	9 (69.2)	3 (23.1)	3 (23.1)	2 (15.4)	0 (0)	0 (0)	1 (7.6)	0 (0)	0 (0)	0 (0)
***p* values**				> 0.05	> 0.05	> 0.05	> 0.05	> 0.05	> 0.05	> 0.05	> 0.05	> 0.05	> 0.05
**Drug consumption**		Yes	73	26 (35.6)	21 (28.77)	7 (9.58)	5 (6.84)	1 (1.4)	1 (1.4)	3 (4.1)	2 (2.7)	0 (0)	0 (0)
		No	106	50 (47.2)	33 (31.13)	8 (7.55)	7 (6.6)	2 (1.9)	1 (0.9)	3 (2.8)	8 (7.5)	4 (3.77)	2 (1.9)
***p* values**				> 0.05	> 0.05	> 0.05	> 0.05	> 0.05	> 0.05	> 0.05	> 0.05	> 0.05	> 0.05
**Drug consuming history (Months)**		1	15	3(20)	3 (20.0)	0 (0)	0 (0)	1 (6.7)	0 (0)	1 (6.7)	0 (0)	0 (0)	0 (0)
		> 1–3	11	3 (27.3)	3 (27.3)	1 (9.1)	1 (9.1)	0 (0)	0 (0)	0 (0)	0 (0)	0 (0)	0 (0)
		> 3–6	25	9 (36)	8 (32)	2 (8)	1 (4)	0 (0)	1 (4)	2 (8)	0 (0)	0 (0)	0 (0)
		> 6–12	15	7 (46.7)	5 (33.3)	2 (13.3)	2 (13.3)	0 (0)	0 (0)	0 (0)	2 (13.3)	0 (0)	0 (0)
		> 12	7	4 (57.1)	2 (28.6)	2 (28.6)	2 (28.6)	0 (0)	0 (0)	0 (0)	0 (0)	0 (0)	1 (14.3)
***p* values**				< 0.05	> 0.05	< 0.05	< 0.05	> 0.05	> 0.05	> 0.05	> 0.05	*p* < 0.0001	> 0.05

## 4 Discussion

This study shows the prevalence, diversity, and associated risk factors for GI parasitic infections in indigenous Tharu people in southern Nepal. The overall prevalence of parasites (42.46%) in this study was lower than in Jalari (*N* = 104, 54.8%) [19], Kumal (*N* = 132, 56.8%) [19], Chepangs (*N* = 100, 97%) [17], Deula (*N* = 150, 68%) [26], Tharu (*N* = 511, 68%) [27], Chepang (*N* = 125, 52%), and higher than in Tharu (*N* = 200, 29.5%) [3], Darai and Kumal (*N* = 189, 25.92%) [28], Chepangs and Musahar (*N* = 205, 36.6%) [29], Meche (*N* = 150, 27.33%) [30], and Magar (31.07%) [31]. Regarding diversity, this study detected nine species of GI parasites (Protozoa: five and Helminths: four) in the Tharu populations compared to previously reported four to fifteen species of GI parasites in various ethnic/indigenous communities from different landscapes of Nepal [3, 17, 19, 26, 27, 28, 29, 30, 31, 32, 33]. In this study, *Ascaris lumbricoides* (5.6%) was present, similar to previous indigenous/ethnic people in Nepal (6.9%–41%) [4, 17, 30, 32, 33, 34]. Second, hookworm was also reported (2.2%), similar to other studies from indigenous/ethnic Nepalese people (1.5%–26%) [4, 17, 30, 32, 33, 34]. Third, *T. trichiura* was reported in this study (1.1%) and from indigenous/ethnic populations of Nepal before (2%–8.3%) [30, 32, 33]. Fourth, a cestode, *Taenia* spp. (3.4%), was recorded similarly to previous studies in Nepal (2%) [30], indicating a reduction in the prevalence of STHs from a few years after deworming programs.

Regarding protozoa, *Cryptosporidium* spp. showed the highest prevalence (30.17%); however, the rate was higher than in Chepang (25%, *N* = 100) [17] and other general populations (*N* = 262, 14.1%) [34], (1%, *N* = 400) [9], (0.79%, *N* = 507) [35], (11.3%, *N* = 9000) [11]. Another coccidian, *C. cayetanensis*, showed a 1.7% prevalence in this study, and it was previously reported in different populations of Nepal (8%–17%) [11, 17, 32, 33]. Both *Cryptosporidium* and *Cyclospora* are foodborne, waterborne, and soilborne apicomplexan parasites [8, 9, 10, 11, 13, 36, 37]; however, the former is transmitted via vertebrate animals or zoonotically and person‐to‐person, whereas the latter does not have such transmission methods [9, 36, 38, 39, 40, 41, 42, 43]. Interestingly, *Entamoeba coli* (8.4%) was also reported in many indigenous groups before from Nepal (2.7%–28%) [17, 30, 32, 34, 44]. Although commensal, it is present in the GI tract that has been invaded by other parasites; therefore, it should not be neglected. *E. histolytica* (8.4%) was also previously found in different indigenous/ethnic populations in Nepal (11%–47%) [17, 32, 33, 34], indicating this Sarcodina parasite is a critical pathogen. Notably, only two fecal samples (1.12%) were positive for *G. lamblia*. However, the prevalence rate was lower than previous reports from Nepal (17%–33.1%) [32, 33, 34], suggesting *Giardia* should be considered during preventive strategies.

In this study, single parasitic infection was significantly higher, although there were a few people with multiparasites. Multiparasitism is clinically critical in enhanced virulence and the absence of host defense [45, 46, 47]; however, mixed infections might be associated with the effectiveness of the antihelminthic drug, including Albendazole, suggesting the significance of concomitance in GI tract [48]. People with *Cryptosporidium* spp. in this study suffered from stomachache or diarrhea, irrespective of concomitance with other parasite/s, suggesting this coccidian can lead to illnesses without the synergistic effects of other parasites. Further investigation is thus required on how *Cryptosporidium* interferes with intestinal physiology and immunology.

Gender did not affect individual, total, or concomitant parasitosis. Interestingly, age groups did not affect total GI parasitosis. In the absence of large sample sizes and unequal total and positive samples among different age groups, it is difficult to explain the age‐wise differences in prevalence rates.

Intestinal parasites are transmitted via contaminated soil, food, water, and air. Their transmission is greatly influenced, established, and enhanced by a few factors like poor socio‐economic status, habits, and behavioral practices of the people and poor surrounding environments [7, 8, 17, 49, 50, 51]. In this population, knowledge of GI parasitosis, diarrhea/stomachache, and drug‐consuming history were associated with total parasites. Similarly, occupation was related to GI parasites; for example, housewives were highly infected with *Cryptosporidium* spp., whereas *E. coli* and *Taenia* sp. were highly present in business people. Also, animal‐husbandry practices affected *Cryptosporidium* species and *Ascaris lumbricoides*. Besides soil, air, water, and food, domestic animals like goats, pigs, buffaloes, cattle, dogs, and cats act as reservoirs for many parasites in these complex landscapes [8, 9, 10, 11, 19, 36, 52, 53, 54]. Cross‐transmission of these species in humans via different domestic animals like cattle, buffaloes, pigs, and dogs might be possible [24, 25, 55, 56, 57]. In addition, farmers usually work with soil, livestock, and the outer environment, and they are always at risk if preventive options are not followed.

Water drinking sources and pork feeding were also risk factors for GI parasitosis. For example, people who drank water from jars or sealed bottles were highly infected with *E. coli and Taenia* sp. although most people used to drink water from handpipes. It is not easy to explain why such discrepancy occurred; it may be possible that not all jar or sealed bottles are free from waterborne intestinal pathogens. Notably, individuals who drank water by treatment were not infected by any parasite, indicating boiling or chlorination is one of the best options for removing parasites [58]. Similarly, pork‐feeding habits increased *Cryptosporidium* species but not all the parasites, including *Taenia* species. There is no transmission route known for *Cryptosporidium* via pork; there exists for *Taenia* species as they complete their asexual stages in the pigs; however, the case study of pork feeding and detection of *Taenia* should be established in the Tharu communities.

In the study area, the majority of the respondents had not consumed antihelminthic (Albendazole) and antiprotozoal (Metronidazole) drugs for more than 1 year. *Entamoeba coli* and *E. histolytica* were statistically higher in the individuals who had consumed drugs before 12 months, raising the necessity of usual treatment by antiprotozoal drugs in the current population. Although not significant, the prevalence of all GI parasites in the drug consumers and non‐consumers was not statistically different, suggesting few possibilities. First, drug resistivity to helminths (Albendazole) and protozoa (Metronidazole) may occur [59, 60]. Second, the above antiprotozoal drugs cannot work against *Cryptosporidium* spp. In addition, these coccidia are not routinely examined in the laboratories, and there is no question of prescription of Nitazoxanide against these coccidia [61] by the health workers at hospitals or community health workers in the local areas. Although drug‐consuming duration showed significant and non‐significant results in different species of GI parasites, such results might be due to low numbers of positive samples in each group. In Nepal, the biannual deworming project for children who studied from class 1 to 5 in 24 districts in 2006 was expanded to 43 districts in 2009 [62]. Then, in collaboration with the United Nations International Children's Emergency Fund (UNICEF), the country conducted a deworming program for children (13–59 months) from December 2017 to 2022 [63]. Notably, a higher rate of nationwide deworming coverage in Madhesh Province compared to the whole country in Kartik 2078 (96.0% vs. 96.7%) and Baisakh 2079 (90.6% vs 91.3%) has been reported by the Ministry of Health and Population, Nepal [64]. Whatever the government reveals deworming coverage rate, GI parasitosis in Madhesh province or Terai region has been predominant in the local population [33, 65]. This might be due to either the deworming program accompanied by the Albendazole administration, which could not control different intestinal parasites, including protozoa and few helminths, or the dominance of risk factors of parasitosis in these populations.

Lack of knowledge on GI parasites had a significant role in parasitosis, although education status did not have any association, indicating awareness rather than education is important in the general population in the rural areas. The symptomatic individuals with diarrhea/stomachache were highly infected with total GI parasites or *Cryptosporidium* spp., despite the similar prevalence rates of other parasites in individuals with or without diarrhea/stomachache. During these symptoms or other GI problems, most people would go to hospitals for treatment, although few would visit medicals or hospitals and witch doctors. In Nepalese society, visiting witch doctors to cure stomachaches and other illnesses is still common, mainly due to their traditional beliefs and lack of awareness of modern medicine.

This study has a few limitations; for example, it was a cross‐sectional study, and we did not perform a case‐control study or others to evaluate risk factors. Second, the laboratory study was solely based on the morphology of the cysts, oocysts, larvae, and eggs of the parasites that might have low sensitivity results. Third, the opportunistic sampling and low sample size might produce a type I and type II error risks. However, we have strength in the research; for example, we have carefully examined the fecal samples by acid‐fast staining and two times by direct wet mount. Also, to cross‐verify, we discussed any unnatural or awkward responses provided by the participant with their family members. Finally, a focal group discussion was conducted to check for any deviation or response bias during the study.

## 5 Conclusions

In conclusion, the research first showed a moderate prevalence and diversity of GI parasites with few risk factors in this study population. Therefore, effective, efficient, timely preventative and control measures should be developed, including raising public awareness of health issues and enhancing environmental cleanliness in this population. In addition, further One Health Approach would be critical by conducting studies that examine samples from the local populations, livestock, food, water, and soil. This will enhance our knowledge of GI parasites' transmission, existence, and epidemiology within this indigenous population, and the control and prevention of such pathogens.

## Author Contributions

P.K.C. participated in the study conception, field study, laboratory analysis, data interpretation, and wrote the first draft of the manuscript. T.R.G. participated in the study conception, statistical analysis, data interpretation, and edited the manuscript, and supervised P.K.C. Both authors have read and approved the final version of the manuscript.

## Ethics Statement

We want to thank for the ethical approval (65/079/080) for this study that was granted by the Institutional Research Committee (IRC), Institute of Science and Technology, Tribhuvan University, Kirtipur, Kathmandu, Nepal. Permission of research was obtained from the Kolhabi Municipality, Bara, Nepal (604/2079/2080). Written informed consent was obtained from each participant.

## Conflicts of Interest

The authors declare no conflicts of interest.

## Transparency Statement

The lead author Pinki Kumari Chaudhary affirms that this manuscript is an honest, accurate, and transparent account of the study being reported; that no important aspects of the study have been omitted; and that any discrepancies from the study as planned (and, if relevant, registered) have been explained.

## Data Availability

The authors confirm that the data supporting the findings of this study are available within the article.
